# Impact Performance Comparison of Advanced Snow Sport Helmets with Dedicated Rotation-Damping Systems

**DOI:** 10.1007/s10439-021-02723-0

**Published:** 2021-02-02

**Authors:** Gina DiGiacomo, Stanley Tsai, Michael Bottlang

**Affiliations:** 1 Biomechanics Laboratory, Legacy Research Institute, Portland, OR 97232 USA; 2 Legacy Biomechanics Laboratory, 1225 NE 2nd Ave, Portland, OR 97215 USA

**Keywords:** Snow sport helmet, Rotation-damping system, Brain injury, Concussion, Oblique impact, Impact testing, Rotational acceleration, Slip liner, Ski, Snowboard

## Abstract

Rotational acceleration of the head is a principal cause of concussion and traumatic brain injury. Several rotation-damping systems for helmets have been introduced to better protect the brain from rotational forces. But these systems have not been evaluated in snow sport helmets. This study investigated two snow sport helmets with different rotation-damping systems, termed MIPS and WaveCel, in comparison to a standard snow sport helmet without a rotation-damping system. Impact performance was evaluated by vertical drops of a helmeted Hybrid III head and neck onto an oblique anvil. Six impact conditions were tested, comprising two impact speeds of 4.8 and 6.2 m/s, and three impact locations. Helmet performance was quantified in terms of the linear and rotational kinematics, and the predicted probability of concussion. Both rotation-damping systems significantly reduced rotational acceleration under all six impact conditions compared to the standard helmet, but their effect on linear acceleration was less consistent. The highest probability of concussion for the standard helmet was 89%, while helmets with MIPS and WaveCel systems exhibited a maximal probability of concussion of 67 and 7%, respectively. In conclusion, rotation-damping systems of advanced snow sport helmets can significantly reduce rotational head acceleration and the associated concussion risk.

## Introduction

Skiing and snowboarding attracted 10.3 million snow sport participants, totaling 59.3 million snow sport visits for the 2018/2019 winter season in the 470 U.S. ski resorts alone.^20^ Over the past decade, helmet adoption has more than doubled to 81^28^ - 90%^13^ of participants in U.S. ski resorts. However, head injury remains the leading cause of death and catastrophic injury in the snow sport population and its incidence rate failed to significantly change in response to increased helmet use.^28^ Of the over 600,000 annual snow sport injuries in North America, 15–20% involve head injuries.^27^ Among snow sport participants younger than 18 years of age, traumatic brain injury (TBI) remains the primary cause in 67% of total fatalities.^15^

Snow sport helmets are the most effective intervention to prevent skull fractures, and they have virtually eliminated scalp lacerations.^31^ A 60% reduction in head injury risk when using snow sport helmets has been reported in a case-control study that employed a non-injured, representative control group to correct for potential confounders.^36^ The U.S. Consumer Product Safety Commission projected that snow sport helmets could prevent 44% of head injuries in adults, estimated at 7,700 injuries annually, and 53% of head injuries of children under 15 years of age, estimated at 2,600 injuries annually.^12^

However, recent advances in helmet design suggest that the effectiveness of helmets may be further improved by targeted mitigation of rotational acceleration of the head.^8^–^10^ Brain tissues are highly susceptible to rotational acceleration of the head, which subjects brain tissue to shear forces that can induce diffuse axonal injury.^16^,^18^,^22^,^29^ Being incompressible, brain tissue has a high resistance to compressive forces associated with linear acceleration, but a very low resistance to shear forces associated with rotational acceleration.^18^ Therefore, rotational acceleration of the head is considered a principal mechanism leading to brain injury.^18^ To account for this unique etiology of brain injury, advanced helmets should be designed and tested for their ability to mitigate rotational forces during real-world impacts.^34^ Given the limited therapeutic interventions to restore neurological function and the increasing awareness of the long-term impacts of concussions, improving the effectiveness of helmet designs to reduce the incidence and severity of brain injury is of critical importance.

Recently, several advanced helmet designs with dedicated rotation-damping systems have been introduced to better protect the brain from rotational forces in oblique impacts.^5^,^9^ The most widely adopted system consists of a slip liner inside the helmet, termed Multidirectional Impact Protection System (MIPS AB, Täby, Sweden), that seeks to reduce rotational acceleration of the head by permitting sliding between the helmet and head during impact. An alternative system employs a collapsible cellular structure (WaveCel, Wilsonville, Oregon) that is elastically recessed inside the helmet to provide a rotational suspension. For bicycle helmets, several designs with dedicated rotation-damping systems have recently been tested for their ability to mitigate rotational acceleration and associated brain injury risk in comparison to standard helmets without a rotation-damping system.^5^,^9^,^10^ Results revealed significant differences in the effectiveness between rotation-damping systems, whereby some systems significantly reduced rotational head acceleration compared to standard helmets, while others did not.^10^ These bicycle helmet results may not be extrapolated to snow sport helmets, which have fewer vents and an inner comfort liner covering the rotation-damping system that may aid or hinder its effectiveness.

The present study evaluated for the first time the effectiveness of rotation-damping systems in snow sport helmets. Standard impact attenuation tests for snow sport helmets do neither induce nor assess rotational acceleration of the head.^19^ Therefore, an advanced helmet impact test method was employed that allowed for helmet testing under oblique impacts to measure rotational headform kinematics and to estimate the associated concussion risk.^9^ Two snow sport helmets with distinct rotation-damping systems were tested in direct comparison to a standard snow sport helmet of similar design without a dedicated rotation-damping system. Testing was conducted at two impact velocities and at three impact locations for each helmet design. Results were used to test the hypothesis that dedicated rotation-damping systems can significantly improve the effectiveness by which snow sport helmets can mitigate linear and rotational head acceleration and the associated concussion risk.

## Materials and Methods

### Helmets

**Figure 1:**
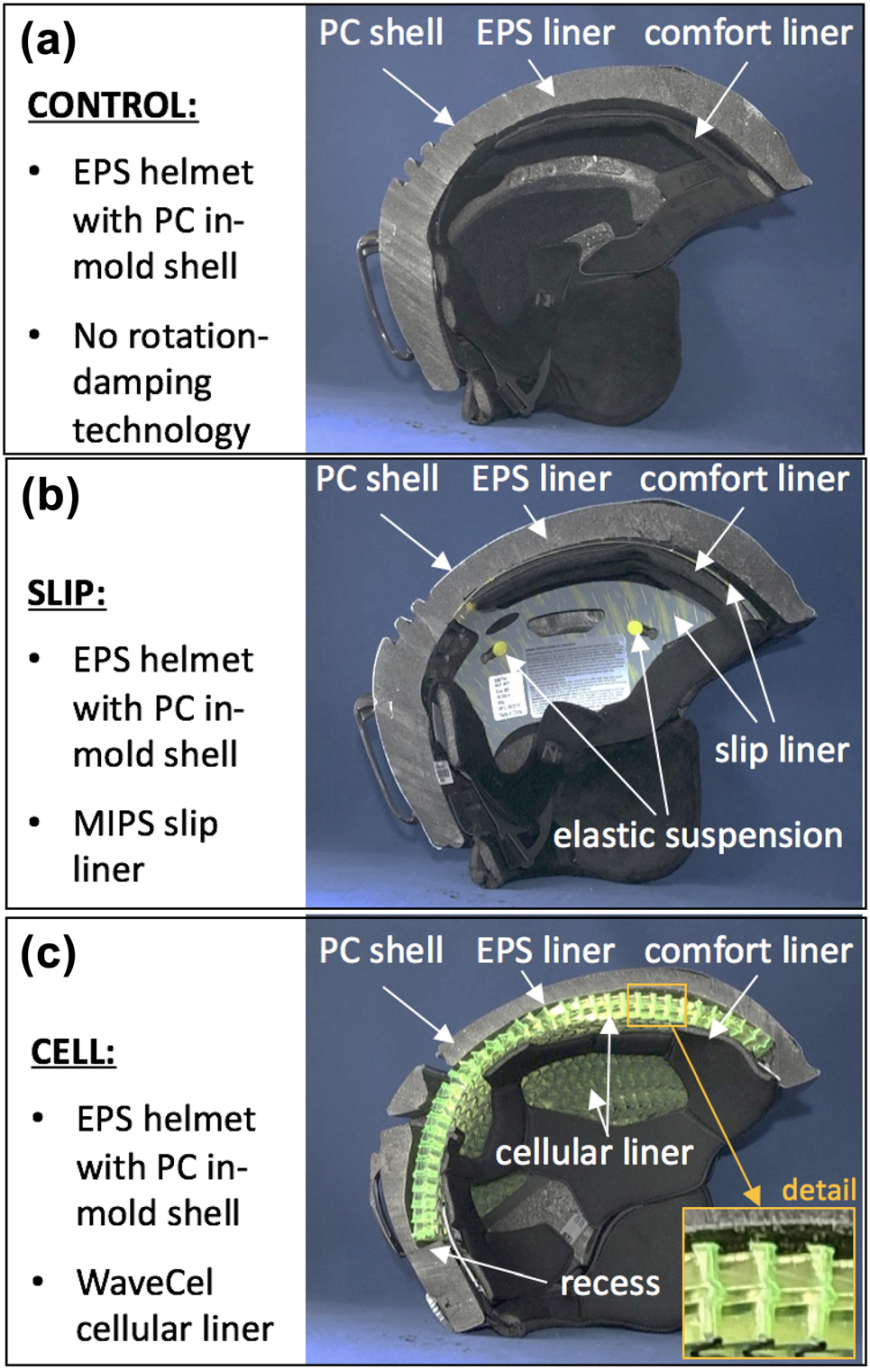
Cross-sectional images of helmets without a rotation-damping system (a), with a slip liner (b), and with a cellular liner (c).

Three different snow sport helmet designs were tested: a control helmet without a rotation-damping system, and two helmets with rotation-damping systems. For the CONTROL group, Smith Maze (www.smithoptics.com) helmets were selected (Fig. 1a). They are standard, mid-range priced snow sport helmets without a dedicated rotation-damping system. The same helmet design with a MIPS slip liner (Smith Maze MIPS) was selected for the SLIP group. The MIPS low friction liner consists of a plastic sheet that is elastically suspended inside the helmet to allow for 10–15mm slip motion between the helmet and head (Fig. 1b). The Anon Logan (www.burton.com) has a WaveCel rotational impact mitigation system that consists of a three-dimensional cellular liner that is recessed inside an EPS shell. Upon impact, the cells of this liner can compress to reduce the impact load and the liner can glide inside its recess to absorb rotational forces. These Anon Logan helmets were selected for the CELL group (Fig. 1c). While the Anon Logan was a different helmet design than the Smith Maze and Maze MIPS helmets, all three helmet designs had an in-molded polycarbonate shell construction which fuses a thin exterior shell with the expanded polystyrene (EPS) liner into a rigid one-piece structure. The thickness of the exterior shell was 0.75 mm for Smith Maze and Maze MIPS helmets, and 0.5 mm for Anon Logan helmets. The total helmet thickness at the front, side, and rear impact locations ranged from 25 to 27 mm for Smith Maze and Maze MIPS helmets, and from 26 to 28 mm for Anon Logan helmets. All three helmets had the same EPS density of 80 gpl and were free of vent features directly at the front, side and rear impact locations. A total of 18 helmets, six per group, were obtained in size medium for testing at the Helmet Impact Testing (HIT) facility of the Legacy Research Institute.

### Test Setup

**Figure 2:**
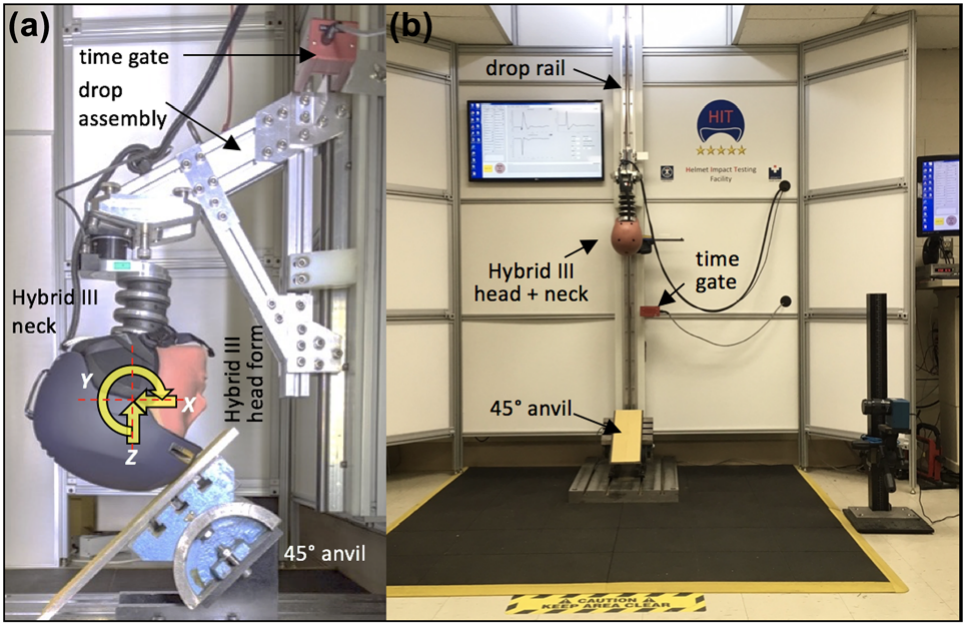
(a) Drop assembly with three linear accelerometers and three angular rate sensors to capture headform kinematics in terms of the resultant linear acceleration (*a*_R_) and rotational acceleration (*α*_R_). (b) Helmet Impact Testing (HIT) facility for vertical drop of Hybrid III head and neck assembly onto a 45° anvil with custom software for real-time data analysis.

Helmet testing was conducted with a Hybrid III 50th percentile male anthropomorphic head and neck surrogate^2^,^4^,^21^,^40^ (78051-336, Humanetics Innovative Solutions, Plymouth, MI) that was connected to a vertical drop tower rail (Fig. 2). A 45° anvil with 80 grid sandpaper was used to induce oblique impacts in response to vertical drops, in line with the impact angle and anvil coating selected by recent helmet comparison studies^7^,^9^,^10^ and the Virginia Tech STAR protocol for helmet testing.^7^ Impact velocity was measured with a time gate (#5012 Velocimeter, Cadex Inc., Quebec, CA). Linear and rotational accelerations of the headform were captured with a six-degrees-of-freedom sensor package (6DX Pro, DTS Inc., Seal Beach, CA) containing three linear accelerometers and three angular rate sensors. This miniature sensor package was mounted at the center of gravity of the Hybrid III head. The resultant linear acceleration *a*_R_ was calculated from the three linear acceleration components. The resultant rotational acceleration *α*_R_ of the headform was calculated by differentiation of the three angular rate signals.

Helmets were tested at two impact speeds, 4.8 and 6.2 m/s. These impact speeds are consistent with the standard specifications for helmets used for recreational snow sports (ASTM F2040-18) for normal impact testing on a hemispherical anvil at 4.8 m/s, and on a flat horizontal anvil at 6.2 m/s.^19^ The 4.8 m/s impact speed onto a 45° impact anvil generated equal tangential and normal impact velocities of 3.4 m/s, whereby a tangential velocity of 3.4 m/s simulates a modest ski or snowboarding velocity of 12 km/h.^3^ Similarly, the 6.2 m/s impact speed onto a 45° impact anvil generated equal tangential and normal impact velocities of 4.4 m/s or 16 km/h. The weight of the drop assembly was 14.3 kg, resulting in impact energies of 166 and 275 J for impact speeds of 4.8 and 6.2 m/s, respectively.

Since the silicone skin surrogate of the Hybrid III headform has over twice the surface friction coefficient of the human head,^38^ a nylon stocking was fitted over the Hybrid III headform to reduce surface friction. This approach was adopted from prior studies that utilized the Hybrid III headform in helmeted drop tests.^10^,^37^,^39^ Helmets were properly fitted to the headform with their original fit system in accordance with the manufacturers’ fit recommendations. Specifically, helmets were positioned with the front rim approximately 7 cm above the basic transverse plane, which intersects the center of the external ear openings and the lower edge of the eye sockets. Retention straps and fit adjustment dials were securely tightened to firmly retain the helmet position during the free fall.

**Figure 3:**
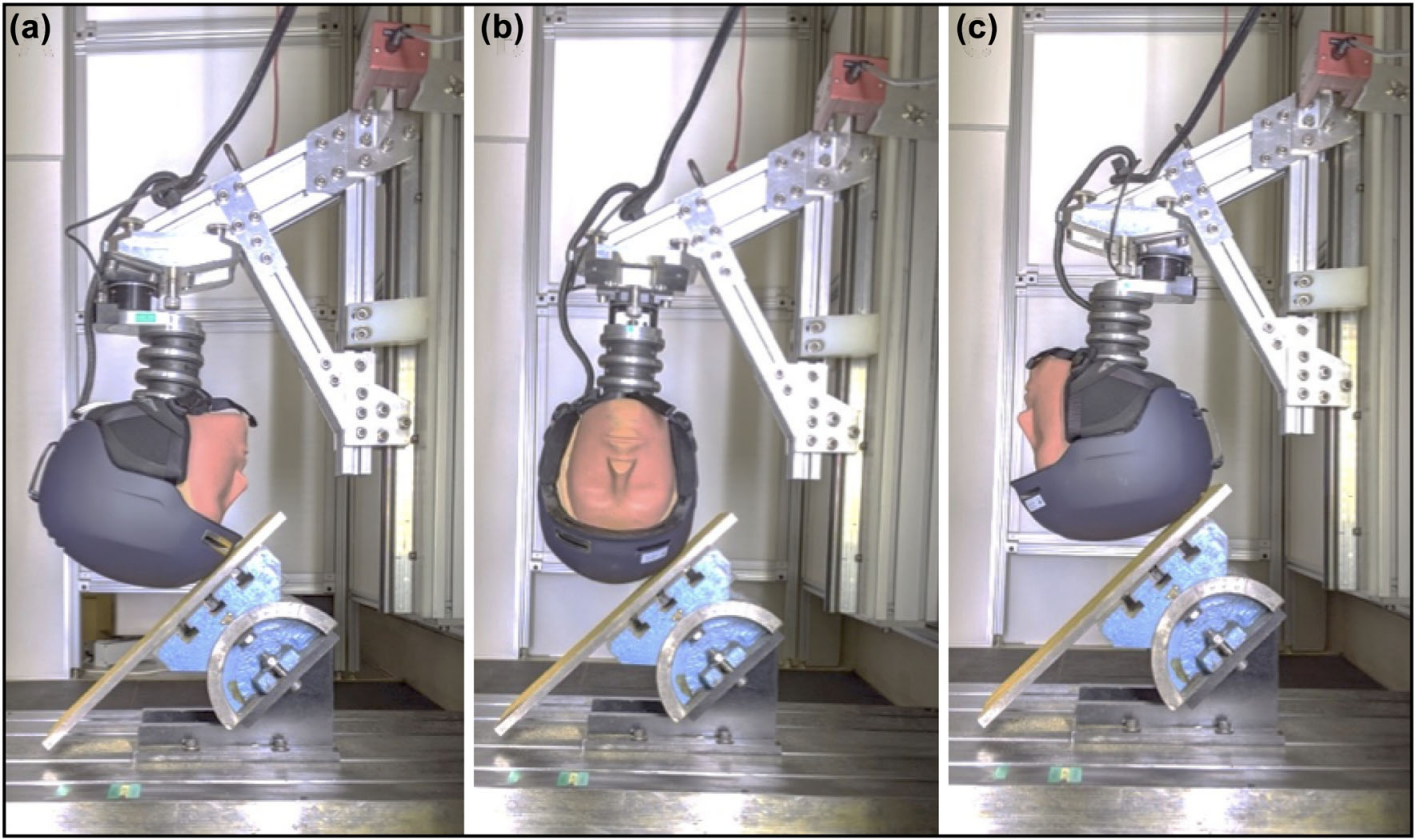
Impact locations at the helmet front (a), side (b), and rear (c) were defined by the alignment of the Hybrid III head and neck surrogate and the 45° impact anvil.

Impact tests were conducted on a front, side, and rear impact location. This was achieved by rotating the base of the Hybrid III neck in 90° increments. The three impact locations were defined by the vertical alignment of the Hybrid III head and neck surrogate and the 45° impact anvil (Fig. 3). Helmets were tested under ambient conditions, defined according to the ASTM standard to be within 17 to 23 °C, and 25–75% relative humidity.^19^

### Data Acquisition and Analysis

Impact kinematics data were captured at a sampling rate of 20 kHz in a data acquisition system (PCI-6221, National Instruments, Austin, TX). Using custom LabView software, linear acceleration signals *a*_*x,*_ *a*_*y,*_ and *a*_*z*_ were low-pass filtered at Channel Frequency Class (CFC) 1000^32^ before calculation of the resultant liner acceleration *a*_R_. Rotational acceleration histories *α*_*x*_, *α*_*y*_, and *α*_*z*_ were calculated by differentiation of rotational rate signals *ω*_*x*,_ *ω*_*y*,_ and *ω*_*z*_, and were used to calculate the resultant rotational acceleration *α*_R_.

The risk of concussion from peak linear acceleration and peak rotational acceleration for a given impact was calculated in terms of the Combined Probability (CP) of concussion (Eq. (1)).^30^ This injury metrics was derived from over 63,000 sports impacts recorded from instrumented football players, and was validated by impact reconstructions of 58 impacts, including 25 concussions, using Hybrid III test dummies.^30^

1 $$ {\text{CP}} = \frac{1}{{1 + e^{{ - \left( { - 10.2 + 0.0433a + 0.000873 - 0.00000092a} \right)}} }} $$

For statistical analysis, headform kinematics (*a*_R_*, α*_R,_ *ω*_R_) and the combined probability of concussion (CP) of the two helmet groups with rotation-damping systems were compared to CONTROL group results, individually for each outcome parameter. Two-sided Student’s *t* tests with Bonferroni correction were used to account for multiple comparisons. A level of *α* = 0.05 was used to detect statistical significance.

## Results

The average speed for low and high velocity impacts was 4.81 ± 0.02 and 6.20 ± 0.03 m/s, respectively. The average energy for low and high velocity impacts was 166 ± 2 and 275 ± 3 J, respectively. There was no statistically significant difference in the average impact speed or impact energy between helmet groups.

### Linear Head Acceleration

**Figure 4:**
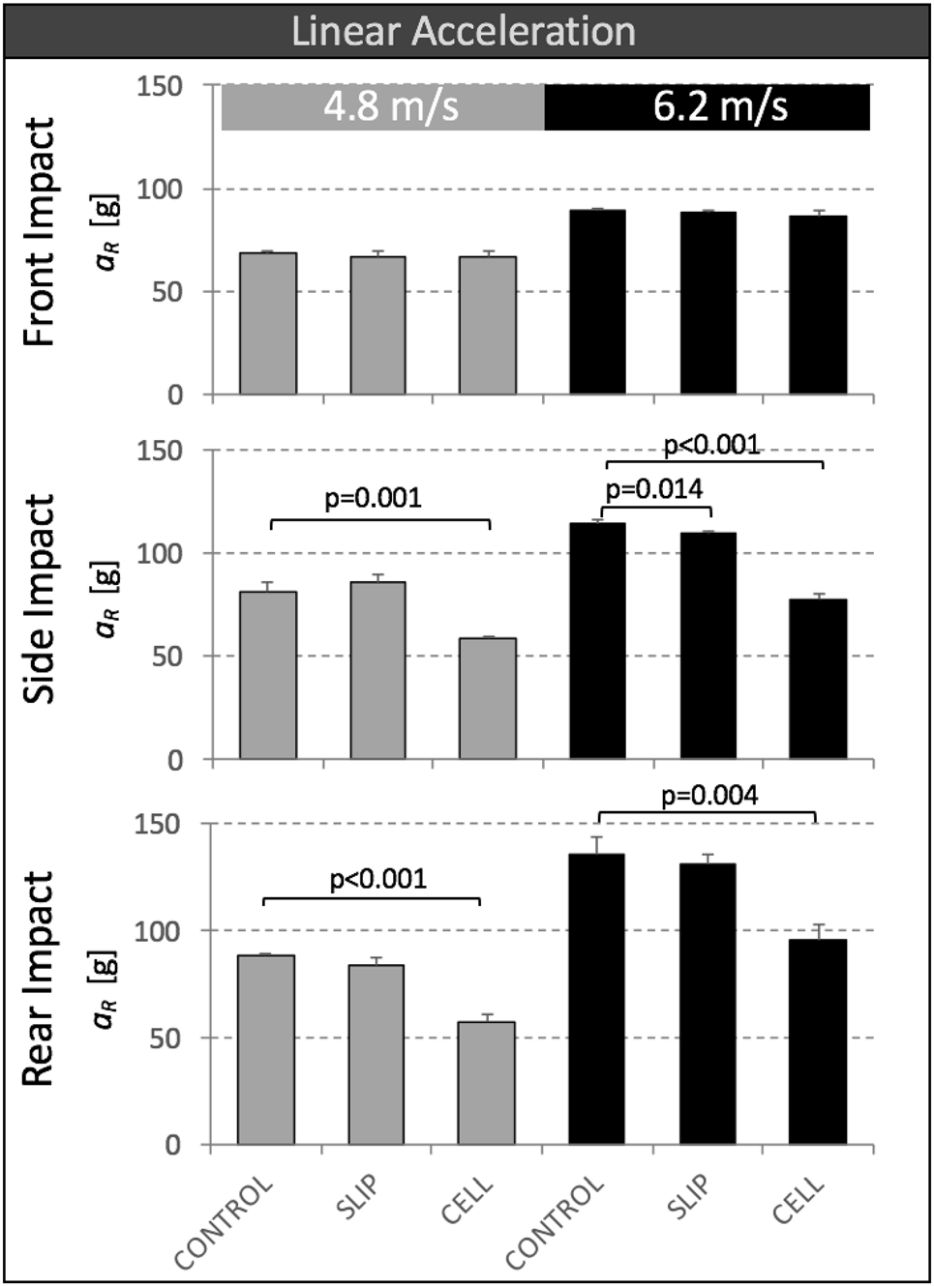
Peak resultant linear acceleration *a*_R_ of CONTROL, SLIP and CELL helmets for impact tests at two speeds and three impact locations.

Peak resultant linear acceleration *a*_R_ of SLIP helmets was not significantly different from that of CONTROL helmets for all six impact conditions, comprising the two impact speeds and three impact locations (Fig. 4). CELL helmets had significantly lower *a*_R_ values than CONTROL helmets in four of the six impact conditions, comprising all side and rear impacts. The highest reduction in peak linear acceleration provided by CELL helmets (36%, *p*<0.001) was observed for rear impacts at 4.8 m/s.

### Rotational Head Velocity

**Figure 5:**
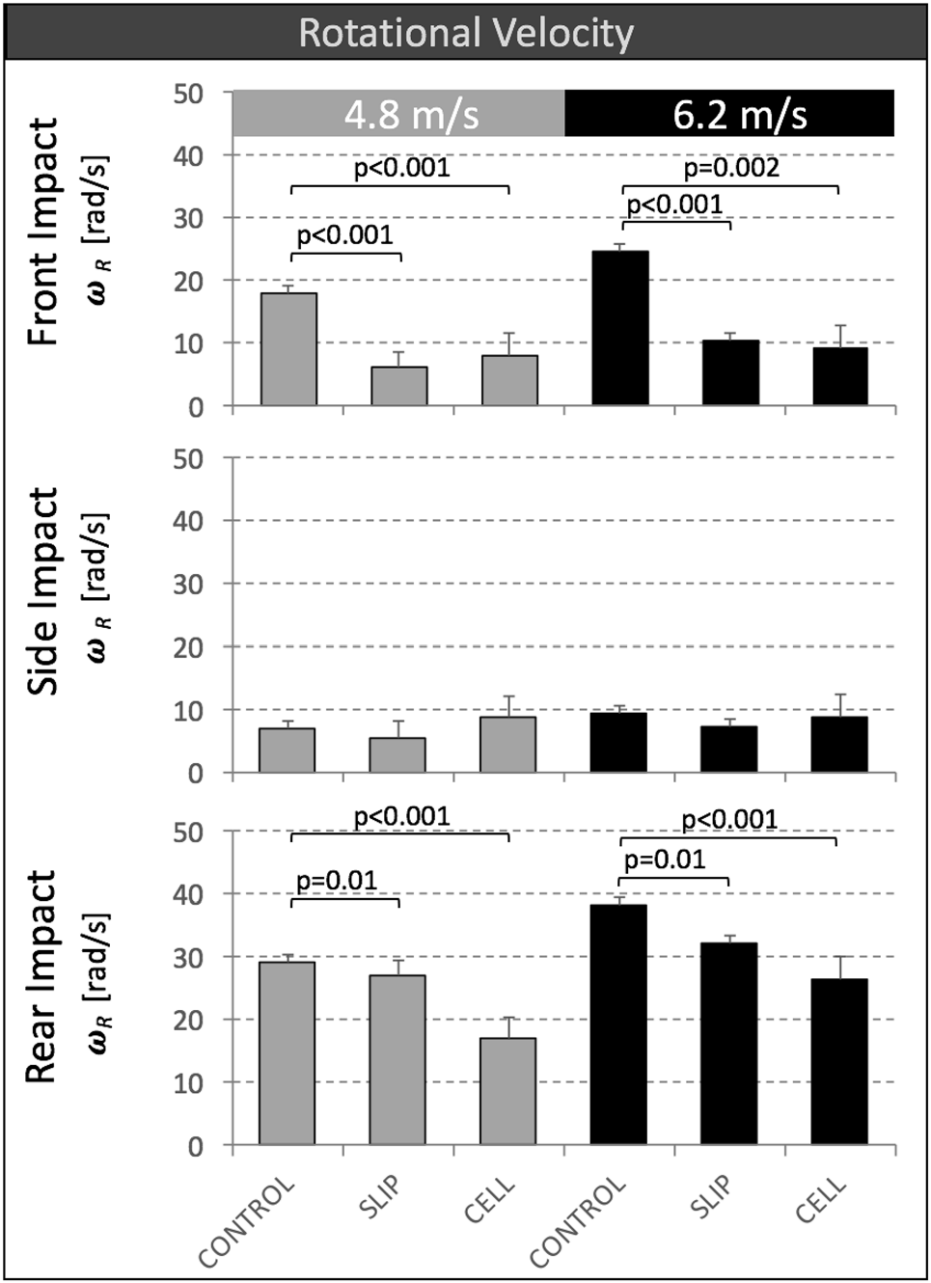
Peak resultant rotational velocity *ω*_R_ of CONTROL, SLIP and CELL helmets for impact tests at two speeds and three impact locations.

Peak resultant rotational velocity *ω*_R_ of SLIP helmets and CELL helmets was significantly smaller than that of CONTROL helmets for all front and rear impacts. (Fig. 5). For these impact locations, reduction in peak *ω*_R_ values provided by SLIP helmets compared to CONTROL helmets ranged from 8 to 66%, with the highest reduction of 66% occurring at front impacts at 4.8 m/s. Reduction in peak *ω*_R_ values provided by CELL helmets compared to CONTROL helmets ranged from 31 to 63%, with the highest reduction of 63% occurring at front impacts at 6.2 m/s. For side impacts, peak *ω*_R_ values remained below 10 rad/s, and there were no significant differences in rotational velocities between groups.

### Rotational Head Acceleration

**Figure 6:**
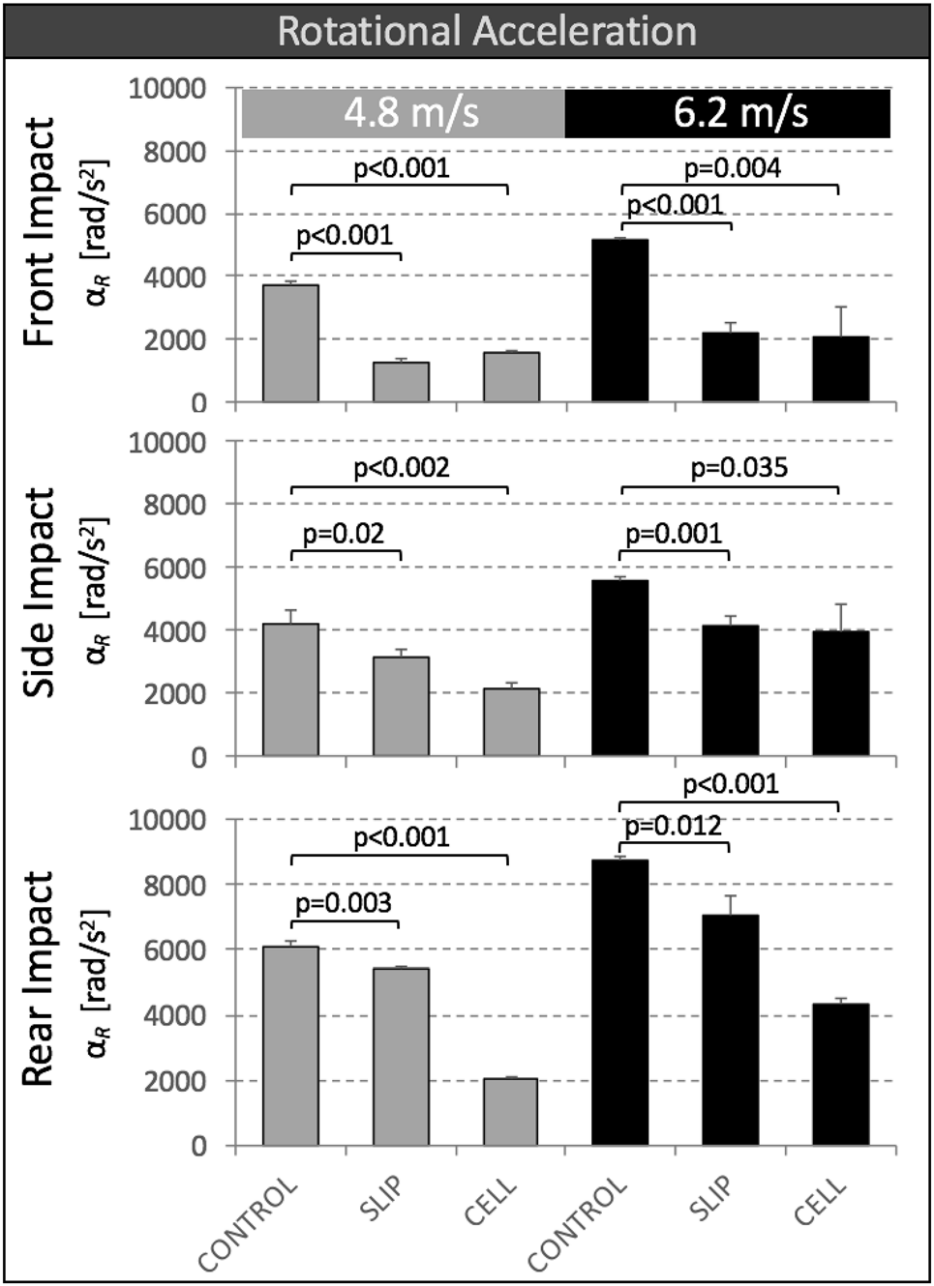
Peak resultant rotational acceleration *α*_R_ of CONTROL, SLIP and CELL helmets for impact tests at two speeds and three impact locations.

Peak resultant rotational acceleration *α*_R_ of SLIP helmets and CELL helmets was significantly smaller than that of CONTROL helmets for all six impact conditions (Fig. 6). Reduction in peak *α*_R_ values provided by SLIP helmets compared to CONTROL helmets ranged from 11 to 66%, with the highest reduction of 66% occurring at front impacts at 4.8 m/s. Reduction in peak *α*_R_ values provided by CELL helmets compared to CONTROL helmets ranged from 29 to 66%, with the highest reduction of 66% occurring at rear impacts at 4.8 m/s.

### Probability of Concussion

**Figure 7:**
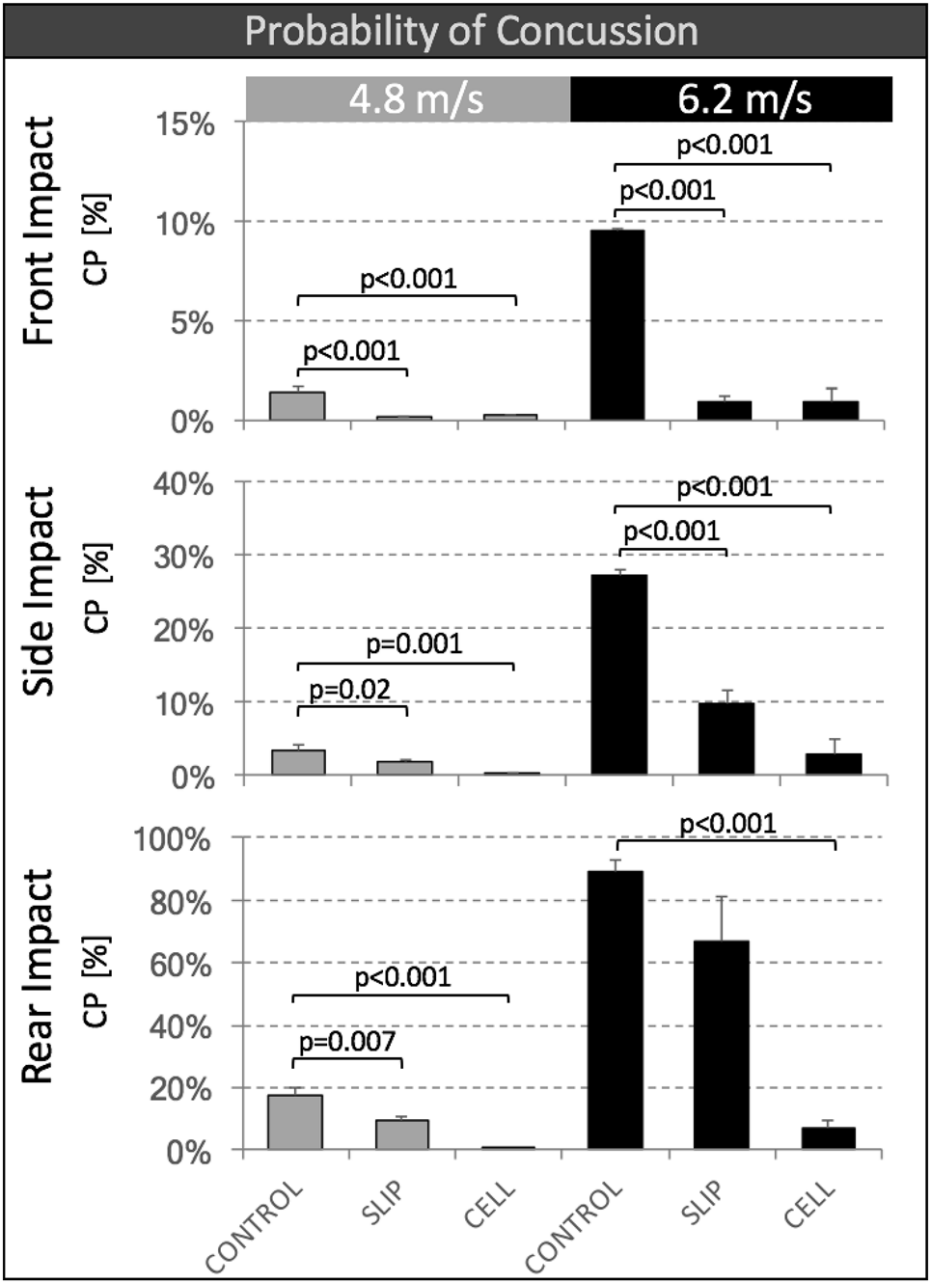
Estimated Combined Probability (CP) of concussion for CONTROL, SLIP and CELL helmets, tested at two impact speeds and three impact locations.

The estimated Combined Probability (CP) of concussion was significantly lower for SLIP helmets compared to CONTROL helmets in five of the six impact conditions (Fig. 7). CP was significantly lower for CELL helmets compared to CONTROL helmets in all six impact conditions. For all three helmet types, CP values were highest for rear impacts at 6.2 m/s. Under this impact condition, CONTROL helmets had a predicted concussion risk of 89 ± 4%. SLIP and CELL helmets reduced the concussion risk to 67 ± 14% (*p*=0.055), and 7 ± 2% (*p*<0.001), respectively.

## Discussion

This research study was able to delineate significant performance differences between advanced snow sport helmets with a dedicated rotation damping system compared to standard helmets that relied only on a rigid EPS foam liner for mitigation of linear and rotational acceleration of the head. Results demonstrated that the dedicated rotation damping systems had no adverse effects on mitigation of linear acceleration and could significantly reduce rotational acceleration and the associated concussion risk.

Peak linear accelerations ranged from 57 to 135 g and remained in all helmets and impact conditions considerably below the 300 g threshold mandated by the test standard for snow sport helmets to prevent skull fractures.^19^ The low friction liner inside SLIP helmets had no effect on linear head acceleration, likely because the 0.5 mm thin plastic sheet is not intended to absorb linear impact forces. Conversely, the three-dimensional cellular structure in CELL helmets likely improved mitigation of linear impact forces by progressive cell compression. Controlled cell compression was aided by a pleat feature in the cell walls of the cellular structure that reduces the force required to initiate cell buckling, similar to an engineered crumple zone. Impact areas in the cellular structure were clearly visible by partial compression of cells.

Peak rotational acceleration, a principal cause of concussion and brain injury,^16^,^18^,^22^ ranged for CONTROL helmets from 3.7 to 8.7 krad/s^2^. A rotational head acceleration of 5.9 krad/s^2^ has been correlated to a 50% probability of sustaining a concussion.^41^ The rotation damping system in SLIP helmets reduced rotational acceleration below this 50% concussions risk threshold for front and side impacts, but not for rear impacts at 6.2 m/s, where SLIP helmets exhibited 7.0 krad/s^2^. At this 6.2 m/s rear impact, CELL helmets also observed their highest rotational acceleration of 4.4 krad/s^2^. Prior studies on bicycle helmets provide varying reports on the effectiveness of SLIP helmets. Bland et al. tested eight standard bicycle helmets and two helmets with a MIPS slip liner in oblique impacts at impact speeds of 5.1 and 6.6 m/s.^5^ While they employed the same Hybrid III neck as the present study, they used a different headform type and orientation, and a 30° anvil. They found that the two MIPS helmet models resulted on average in 6.0 krad/s^2^ peak rotational acceleration, while the eight helmet models without a MIPS slip liner resulted in a lower peak rotational acceleration of 5.3 krad/s^2^. Another study employed the same test conditions as in the present study and reported for 6.2 m/s front impacts a significantly lower peak rotational acceleration of 5.7 krad/s^2^ for bicycle helmets with a MIPS slip liner than for the same helmets without a slip liner (7.2 krad/s^2^).^10^ Under the same test condition, the present study reported a 2.2 krad/s^2^ peak rotational acceleration for snow sport helmets with a MIPS slip liner. This impressive result suggests that the effectiveness of slip liners depends in part on their integration inside a helmet. SLIP helmets in the presents study had a soft felt lining glued to the inside of the EPS shell, presumably to facilitate sliding of the slip liner during impact. Furthermore, the slip liner was captured in a comfort liner, which also may have improved its performance compared to the previously tested bicycle helmet with a MIPS slip liner. Since the performance of slip liners likely depends on helmet design, the considerable performance improvement observed with SLIP helmets in the present study may not be readily extrapolated to other helmets with slip liner technology.

The combined probability of concussion takes into account both peak linear and rotational acceleration histories.^30^ The highest concussion probability of 89% was observed for CONTROL helmets at 6.2 m/s rear impacts. At this impact condition, SLIP and CELL helmets reduced the concussion risk by 22 and 82% respectively. This considerable reduction of concussion probability with CELL helmets is consistent with a prior study on front impacts of bicycle helmets with CELL liners.^9^ Bicycle helmets with CELL liners of the prior study and CELL snow sport helmets in the present study both yielded a 1% concussion probability for 6.2 m/s front impacts. A recent study further confirmed a positive correlation between impact testing and real-world performance of helmets, whereby helmets which exhibited reduced headform accelerations in laboratory tests were also associated with lower concussion rates.^1^ However, predicting an absolute concussion probability depends on the accuracy of injury risk curves that have been reconstructed from a limited number of real-world injury data to estimate brain tolerance limits. Moreover, these injury risk curves are highly non-linear, for which reason a relatively small difference in peak rotational velocity can translate into a large difference in injury probability.^5^ The uncertainty in defining brain tolerance limits combined with the non-linear nature of injury risk curves necessarily limits the accuracy in predicting an absolute probability of concussion. However, relative differences in concussion probability between helmet technologies should provide a meaningful comparison, since the helmet technologies were tested in similar helmet models under defined and reproducible impact conditions.

In standard test methods, rotational acceleration of the head is neither induced nor measured.^19^ The present findings emphasize the need for advanced impact testing of snow sport helmets under impact conditions that capture linear as well as rotational head acceleration and associated concussion risk. Such testing will be critical to guide developers towards the design of more effective rotation-damping systems, and to educate consumers on helmets that provide the best concussion protection. Several researchers have previously compared the performance of helmets in oblique impacts.^5^,^9^,^10^,^25^,^35^ At present, the main resource to compare helmet performance in oblique impact testing is provided by the helmet laboratory at Virginia Tech University. They analyze linear acceleration and rotational velocity of the headform to derive a Summation of Tests for Analysis of Risk (STAR) score and a star rating, ranging from 0 to 5 stars.^7^ Virginia Tech’s 5-star rating has been a driving force motivating manufacturers to consider rotation-damping systems. To date, they have tested helmets for a wide range of sports and outdoor activities, but not for snow sports.

Results of this study are limited to a specific test configuration and may not be extrapolated outside these test parameters. Results are specific to impacts onto a 45° anvil covered with 80 grid sandpaper for consistency with precedence of prior studies.^5^–^10^,^17^ A pilot study was conducted to explore the effect of sandpaper by impacting six additional Smith Maze helmets onto a 45° anvil with a polished steel surface without sandpaper at 4.8 and 6.2 m/s at front, side and rear impact locations. Averaged across all impact scenarios, impacts without sandpaper yielded the same linear acceleration, a 9% lower rotational velocity, an 18% lower rotational acceleration, and a 16% lower probability of concussion than impacts with an 80 grid sandpaper. Results are also specific to a Hybrid III 50th percentile male anthropomorphic head, which was chosen because it is the most widely used human head surrogate employed for impact testing.^4^ It provides an elastic skin envelope, and its inertial properties are considerably more biofidelic than those of ISO headforms specified in the CPSC safety standard.^40^ While the headform of the National Operating Committee on Standards for Athletic Equipment (NOCSAE) is considered to have the most biofidelic headform shape, integration of a neck and instrumentation is more difficult compared to a Hybrid III head.^11^ While there is precedent for impact testing using an unconstrained headform without a neck surrogate,^14^,^23^,^25^,^26^ the present study simulated quasi-physiologic head constraints with a Hybrid III neck.^5^ The Hybrid III neck was validated for flexion and extension, but has been shown to be overly stiff in lateral bending and axial compression.^33^ The Hybrid III head and neck combination has been used in a range of helmet impact studies^4^,^5^,^17^,^24^,^29^ and has been proposed for advanced testing of helmets.^40^

A recent study by Bland *et al*. evaluated the effects of the Hybrid III neck in oblique impacts onto a 45° anvil at 6 m/s.^6^ Using only a Hybrid III headform without a neck produced 17–35% greater peak linear and rotational accelerations than tests with the Hybrid III neck. While their results demonstrated that a neck does effect impact kinematics despite the considerably short time scale in the 10 ms range, the effects of the neck were inconsistent and differed between front and side impacts. While their finding should be considered when comparing the present results to studies that did not use a neck, it does not detract from the validity of relative comparisons with the present study. As baseline for a conventional snow sport helmet, only a single control model was tested. Considerable variability in impact performance is to be expected between conventional snow sport helmets with different shell and EPS liner designs. Hence, the relative benefits of helmets with rotation-damping systems described in this study are limited to the single control model tested. Finally, the present study relied on the tangential velocity component during an oblique impact to induce rotational head acceleration. However, the head can also exhibit rotational forces from a normal, non-oblique impact to the side or back of the helmet, which causes the head to rotate around the lower neck. This is particularly relevant to snowboarding, whereby the most impacted area of the head is the back or occiput (53%), caused by backward falls induced by the back edge of the snowboard catching the slope.^3^

In conclusion, results demonstrated that rotation-damping systems of advanced snow sport helmets can significantly reduce rotational head acceleration and the associated concussion risk. These results emphasize the need for advanced impact testing of snow sport helmets that considers linear and rotational head acceleration to enhance the effectiveness of helmets in reducing the incidence and severity of concussion and TBI.
